# Supplementary material for: Insights from the care home staff on the use of observational risk assessment of contractures: Longitudinal evaluation (ORACLE): A qualitative study
Source: PLoS One. 2025 Oct 31;20(10):e0335658. doi: 10.1371/journal.pone.0335658 (PMC12578150; doi:10.1371/journal.pone.0335658)
Supplement: S1 Appendix — (DOCX) [file pone.0335658.s001.docx]

**Topic Guide for Interviews (Care Home Managers/Senior Staff)**

1. **Introduction, welcome and demographics (age, gender, experience, speciality etc)**

- Tell me about your experience of ORACLE use in the care home?
- Did anything work well in the tool?
- Is there anything that did not work well in the tool?
- Tell me about how you found the wording and questions in the tool.
- Tell me about your experience with the recommendations based on the level of risk in the tool.
- Tell me about your experience of the training session? Is there anything you would change or want more information about?

1. **Feedback about the layout, and language of the tool?**

- How did you find the wording of the tool?
- Ease of use – scoring, time taken to complete the observation etc.
- Overall layout of the tool (font, font size etc).
- Prompts
  - Can you tell me more about that?

1. **Practical implementation of ORACLE**
   - Do you have the responsibility for organising the day today routine of the residents?
   - If you do, how easy or difficult it was to incorporate the tool into their routine?
   - How are the in-reaching services (e.g. PTs and OTs, GP, spasticity management services etc.) supporting you in response to the referrals made through this tool?
   - Does the care home have the appropriate resources (time, staff & skills) to regularly perform assessments?
   - Education and training needs of the staff to use the tool effectively.
   - Prompts
     - Can you tell me more about that?
2. **Contextual factors that need to be considered to optimise the usability of the tool within a care home**
   - Nutrition/Diet
   - Medications
   - Opportunity to engage socially with friends, family, and other community members
   - Support from family, healthcare professionals etc
   - Prompts
     1. Can you tell me more about that…?
3. **Ending**

- Is there anything else you would like to add or talk about anything we have not discussed?
- Thank you so much for your valuable time.

**Topic Guide for Interviews (Healthcare Assistants)**

1. **Introduction, welcome and demographics (age, gender, experience, speciality etc)**

- Tell me about your experience using the tool.
- Did anything work well in the tool?
- Is there anything that did not work well in the tool?
- Tell me about how you found the wording and questions in the tool.
- Tell me about your experience with the recommendations based on the level of risk in the tool.
- Tell me about your experience of the training session? Is there anything you would change or want more information about?

1. **Feedback about the layout and language of the tool?**

- How did you find the wording of the tool?
- Ease of use – scoring, time taken to complete the observation etc.
- Overall layout of the tool (font, font size etc).
- Prompts
  - Can you tell me more about that?

1. **Completing the tool**

- When did you find the best time to complete it?
- How easy or difficult it was to incorporate the tool into your work routine?
- Discussion around training needs
- Prompts
  - Can you tell me more about that…?

1. **Ending**

- Is there anything else you would like to add or talk about anything we have not discussed?
- Thank you so much for your valuable time.
