# Supplementary material for: Insights from the care home staff on the use of observational risk assessment of contractures: Longitudinal evaluation (ORACLE): A qualitative study
Source: PLoS One. 2025 Oct 31;20(10):e0335658. doi: 10.1371/journal.pone.0335658 (PMC12578150; doi:10.1371/journal.pone.0335658)
Supplement: S2 Appendix — (DOCX) [file pone.0335658.s002.docx]

| **Themes** | **Example Quotations** |
| --- | --- |
| **Theme 1: Usability** | |
| ***Sub-theme: ease of use*** | *‘Well, I think the tool is quite user-friendly…even the new staff found it straightforward once they got the hang of it’* |
|  | *‘The scoring was very easy…Just add them all up, and it's straightforward’* |
|  | *'It was easy to understand. I think it was well set out, and easy to complete…and I think the carers were all quite comfortable using it as well…as if they'd sort of done it before, which was quite good'* |
|  | *‘Yeah, I think to be honest, it's been relatively straightforward. You know the tool itself is quite self-explanatory and it's just a case of getting the carers to complete it, which I think because we know our residents and you know their room with them every day…Yeah, it's quite straightforward’* |
|  | *‘I think it's really easy to use… easy to follow… and easy to understand’* |
|  | *‘I think I found that one like quite easy and simple to understand and anyone can do it once they have like knowledge of that… It's quite handy actually’* |
|  | *‘The tool was easy to understand and complete. The carers seemed comfortable using it as if they'd done it before’* |
|  | *‘..the numbers, for example, I think are nice, like 0, 1, 2, because it makes it simple’* |
| ***Sub-theme: layout and language*** | *‘It’s laid out in a way that makes it easy to follow’* |
|  | *‘The language used was clear and easy to understand, and I liked the overall layout of this tool…yeah it was easily readable’* |
|  | *‘Yeah, because you've got that big. So even when moving the glasses off, I can't read the big letters. But yeah, that'd be fine’* |
|  | *‘The wording is very good… I could understand it perfectly’* |
|  | *‘The tool was set out well’* |
|  | *‘Not really any issues [with the language and layout]’* |
|  | *‘Yeah, because you've got that big. So even when moving the glasses off, I can't read the big letters. But yeah, that'd be fine’* |
| ***Sub-theme: time efficiency*** | *‘This would probably take a couple of minutes because when you've been with them all day… you know what they're doing…you know what they're not, so you just quickly put them in the category they are...which wouldn’t take long’* |
|  | *'Actually, it's been a really long time that I've been working here, so I know the residents here very well. So, for us, it doesn't take that long out there because we are working with them in day-to-day life. So, it only took a couple of minutes for one resident. But yeah, it might not be the same for new or agency staff'* |
|  | *‘It doesn't take too long. That's the nice thing’* |
|  | *‘It only takes like a couple of minutes’* |
|  | *‘For one resident?..I don’t know…may be 2-5 minutes’* |
| ***Sub-theme: challenges in assessment*** | *‘Where it says if they are able to carry out activities... it was a little bit difficult to judge because some of them can eat themselves but might not be able to dress. So, I feel like maybe there could be a bit of separation or something’* |
|  | *'It's really hard to put them into a zero, one, or two because some of them will dress partly, eat partly. You know, some will eat the main meal but then can't eat the pudding. It depends on the cutlery and the bowls. I struggled because some of them could dress their top half but not their lower half. So, yeah, I struggled to categorise them in that box'* |
|  | *‘A lot of them can't really tell you anything… so that was a bit of a judge…I was like, oh, no pain, but maybe there is pain because people with dementia can't really express it. So, that will be one that was a little bit difficult to judge’* |
| **Theme 2: Acceptability** | |
| ***Sub-theme: integration into routine*** | *'You're doing their charts anyway in their room… with this, you could just gauge down to check nothing's changed, and that's it. Incorporate it in when they do the daily records. And then you could do this one as well and do a risk assessment, so you'd know if anything's changed'* |
|  | *'Day-to-day it would be quite difficult because every day is not the same. Sometimes we can't even spare a couple of minutes. But once a month or once every couple of weeks, it would be fine' (Carer #4)* |
|  | *'Probably after they've got up in the morning and we've done all the care…. then we could go through this to make sure if things have changed.. because we would have seen all their body…we would have moved them... we would have done all that is required and spoke to them so then that would be easy to fill out'* |
|  | *'Morning and evening are quite busy for us, so anytime in mid-afternoon'* |
| ***Sub-theme: quality of care*** | *'It covers important areas like mobility, hydration, nutrition, pain, and skin health. It all links together to give us a complete picture of the resident's risk and improve their care accordingly'* |
|  | *‘…and at the end of the day, if it comes into a tool and we start using it, I think it will be very helpful for preventing, which is like the best medicine, isn’t it?’* |
| ***Sub-theme: behavioural changes in practice*** | *'It is harder to deal with contractures than it is if they can move their legs around… because we used to have a lady, her legs were stuck…well, you can't get to do personal care…you can't do nothing because you can't force the legs…once they've developed... So, now we try and get them to at least try and move their leg. Or if they can't, we move them so that at least the muscles still moving'* |
|  | *'We've had sort of recent recruitment where we've had new people come in…So I have kind of earmarked it to sort of go through with them and just give them a rough outline because I don't want to make it too complex for them'* |
|  | *I think using it has made us realise how much contractures actually occur and how vulnerable they are, and how many we actually do have in here that aren't actually really dealt with that much.* |
|  | *‘We use it mainly when someone's just come in, mainly so we can gauge whether they're at high risk for contractures, what exercises they need and different things. And people that are not getting up now. So we check them, make sure they've got the movement so they don't get contractures… so we use this like to see if they can move every day, really’* |
|  | *‘Also this happened to my mother, who has passed away because they didn't move her... they just left her in bed…and of course, the legs creep up because the muscle just stiffens and can't do anything though. So I'm more aware of keeping them moving…and this helps because you've got each step. and obviously if they're in pain, then we can increase pain relief so that they can move the legs’* |
| ***Sub-theme: suggestions for improvement*** | *‘I do feel it does need like a network of people to come together. I do feel it's quite a lot for, say, one or two people to do sort of independently. If a group of people, you know, get together and just work on the actual tool itself. I think that would definitely be better to apply the tool in routine care’* |
|  | *'More training, I was just about to say. Yeah, giving us more training would be handy'* |
|  | *'I've never seen that in any agency staff ever talk about contractures, so there's a missing link there, definitely'* |
| **Theme 3: Contextual Factors** | |
| ***Sub-theme: Dementia*** | *'I think that would definitely be a useful tool with some tips on how we could facilitate that with our dementia residents.* *Maybe that could be displayed in sort of visual cards, you know, explaining what we want to do. It's very difficult sometimes to get that engagement, you know, and get them focused on the task you want them to do'* |
|  | *‘..they won't follow instructions, they'll just rely on you for literally everything... umm, they can't understand anything. they can't move themselves…so it's you who is doing all the jobs…. if you help exercise them, I think you could reduce a lot of the contractures’* |
| ***Sub-theme: nutrition and hydration*** | *'So we had a lady [resident] diagnosed with a type of dementia which affected her perception of food, which caused her to stop eating. In the last six months, she started eating again but not full meals, only biscuits and things like that., she gained weight which affected her ability to turn as well'* |
|  | *'If they're not hydrated, they are going to sleep more…they're going to move less and therefore, we're doing them a disservice... and obviously then, that impacts their skin integrity. …and if their skin integrity is affected and they've got pressure sores again, they're going to be less likely wanting to move…they might be in pain'* |
|  | *‘Nutrition is crucial. We have residents with PEG feeds, those who might gain too much weight affecting their mobility, and others who need to maintain a balanced diet to stay active’* |
|  | *‘I just think that you can always tell when a resident is well, you know, and just like the lack of hydration, even with like you and me, if we don't drink water, you know we're just not on our best form and I think it's the same with residents, you know… if you're not hydrated, they are going to sleep more…they're going to move less and therefore, we're doing them a disservice.. and obviously then, that impacts their skin integrity…. and, if their skin integrity is affected and they've got pressure sores again, they're going to be less likely wanting to move. they might be in pain. So yeah, its massively connected’* |
|  | *‘Proper hydration and nutrition are important… for them to be mobile and… because always if they're like dehydrated, all they will do is sleep all day... they don't eat, they haven't got the energy. So, I think that is a really important fact’* |
|  | *‘Yeah, we have had that in the past..they became too overweight and then they've just given up doing anything for themselves…they just stopped walking’* |
| ***Sub-theme: medications*** | *'You know, if somebody is on furosemide and they're needing the toilet a lot and they're having to get up and go, ok, they're moving… but equally, they probably just want to sleep... they're probably tired. So, they're probably sleeping, toilet sleeping, you know, and again then they might not be eating and drinking as well because they're not having a good, peaceful sleep. So yeah, it has an impact'* |
|  | *'…,we have residents who are on medications to help with any sort of aggressive behaviour or if they've been anxious. But it also means that they become sleepy and if it's somebody who is quite mobile, then they're at risk of falls. So, getting the timings right for their medications is important. Otherwise, if they fall, then we're going to have problems because that will, you know, have an effect on their mobility'* |
|  | *‘Medications like antipsychotics can make residents drowsy..which increases their risk of falls… It's important to get the timing right for medications to avoid these issues’* |
|  | *‘The medication makes her tired and it's kind of like you can just see it is a downward spiral and there's nothing really to support’* |
| ***Sub-theme: motivation and engagement*** | *‘We have some people like that in the activities class... They just like, didn't want to eat….they don't want to drink…they don't want to do nothing..’* |
| ***Sub-theme: family/friends/ peer support*** | *'You can see the families where the residents whose families come again; it gives them that sense of purpose. Again, the families are also providing their care needs, like, even if it's just, they're coming to talk to them it then it frees the carers up to be able to go and spend time with those other residents that maybe don't have the family and to be able to do the things to help them'* |
|  | *'Yeah definitely, even if we're talking, we talk with our hands, don't we? And just moving… its all the little things even if that's anything like getting someone's attention, Oh Hello! And things like that... compared to not having any reason to move'* |
|  | *‘It's very important.. our well-being team ensures there are always activities. Family visits and social interactions give residents a sense of life and purpose, encouraging them to stay active’* |
|  | *‘Yeah, it is important because it's just about the whole. It's the holistic approach, isn't it?’* |
|  | *‘I think they should get more involved, definitely… if it were my mum, I would want tha’* |
| ***Sub-theme: consistency of care*** | *'.. I think in general across the industry, there is a lack of time and lack of staff…like for example we had a lady [carer] who left us, and she's gone to work in another care home, and she said, so far all I've done is work with agency staff. So, there's inconsistency of care, the staff not knowing the care plans, the staff not knowing the residents'* |
| ***Sub-theme: specialist support*** | *'I feel like it could be improved. One of the recent ones [physio] was quite good because he actually put some pictures of how he wants us to position the resident and then the pillows, and that has been very handy in comparison with other times that they've come and they just talk to the nurse. We're the ones who do their personal care, so the information gets lost, you know?'* |
|  | *‘In general, I think here …we are really really lucky.. I know that when we do make referrals anywhere, we tend to get results pretty quickly, like sometimes within a couple of days.. might be a couple of weeks, but we are generally quite lucky’* |
|  | *‘…even if we sent a referral and we didn't get an answer in most places we were able to give them a ring and maybe they can advise over the phone and they can say we'll look for the interim, these would be our recommendations, but you know we've got an appointment scheduled or we will get an appointment scheduled and we'll come and see the person. So yeah, we do tend to get quite good support’* |
|  | *‘To be honest. I do think, in general, I feel like in care homes they could have more occupational therapy, like physios that come in more often. It would really benefit these places because we can do only so much..and I feel like if they come more often, it would really help’* |
|  | *‘When they come, they do give us this kind of, you know, exercises to follow for some of them, but still… some people can do it. But I feel like sometimes, if am I doing it right because I don’t really know what I’m doing. Even if someone explains it once to you, you know’* |
| ***Sub-theme: staffing and workload*** | *‘Yeah. I think in general across the industry, there is a lack of time and lack of staff’* |
|  | *‘That [having more staff] would always be a solution. But we know that's never going to happen.. some of them do get ignored unintentionally because of workload….especially ones that can’t communicate with something’* |
|  | *‘I do believe having the extra hands on the days we have extra people does make a difference in everything, like for them and us…yeah it reduces your burden’* |
|  | *‘If the carers are busy, you do sometimes think that some residents get more support than others… like, in our dementia unit, it’s quite even. But when you go into a residential setting, the honest truth is, it’s often whoever shouts the loudest. You might have a resident who’s always on the buzzer, like, ‘I need this, I need that.’ A classic would be someone asking about dinner…like, it’s in two minutes..but the carers are busy, you know?’* |
| ***Sub-theme: training and regular updates*** | *‘No, there's not much training for contractures. We have loads of training, but nothing specifically for contractures’* |
|  | *‘Staff need to recognise deterioration, pain, and how to safely carry out exercises’* |
|  | *‘For contractures, we have had one before.. they were really bad. We sent out some paperwork…. Training help the resident stop being in pain and reduces the need for medication’* |
|  | *'More training, I was just about to say. Yeah, giving us more training would be handy'* |
|  | *‘They know people can get contractures, but they don't know the ins and outs and how they can improve it…So yes, that's the only one that's not covered in training. Because they train manual handling, they do everything else but not contractures’* |
