# Supplementary material for: Insights from the care home staff on the use of observational risk assessment of contractures: Longitudinal evaluation (ORACLE): A qualitative study
Source: PLoS One. 2025 Oct 31;20(10):e0335658. doi: 10.1371/journal.pone.0335658 (PMC12578150; doi:10.1371/journal.pone.0335658)
Supplement: Attachment — Submitted filename: Response to Reviewers.docx [file pone.0335658.s004.docx]

**Response to Editor and Reviewers**

12/06/2025

PONE-D-24-50857

Dear Editor,

Dear Reviewers,

We would like to thank you for the opportunity to resubmit the revised copy of the paper, ***“Insights from the Care Home Staff on the Use of Observational Risk Assessment of Contractures: Longitudinal Evaluation (ORACLE): A Qualitative Study”***

We really appreciate your constructive and valuable feedback. We hope we have addressed your concerns to your satisfaction. Please find below our responses to each of the points raised.

Best regards ,

Hina Tariq

**Response to the academic editor**

1. Please ensure that your manuscript meets PLOS ONE's style requirements, including those for file naming. The PLOS ONE style templates can be found at

[https://journals.plos.org/plosone/s/file?id=wjVg/PLOSOne_formatting_sample_main_body.pdf](https://eur02.safelinks.protection.outlook.com/?url=https%3A%2F%2Ftrack.editorialmanager.com%2FCL0%2Fhttps%3A%252F%252Fjournals.plos.org%252Fplosone%252Fs%252Ffile%253Fid%3DwjVg%252FPLOSOne_formatting_sample_main_body.pdf%2F1%2F010f019593e6ecbc-9921ebc3-6552-4ce5-bf9e-bb85feb80e26-000000%2FG68e26Xr2YgmgnlteXqSvL_K4UfoAq9RpDjF0skZV2M%3D202&data=05%7C02%7Chtariq%40bournemouth.ac.uk%7C7bc3187e8f01447e8ddd08dd80e78192%7Cede29655d09742e4bbb5f38d427fbfb8%7C0%7C0%7C638808454268678849%7CUnknown%7CTWFpbGZsb3d8eyJFbXB0eU1hcGkiOnRydWUsIlYiOiIwLjAuMDAwMCIsIlAiOiJXaW4zMiIsIkFOIjoiTWFpbCIsIldUIjoyfQ%3D%3D%7C0%7C%7C%7C&sdata=yNMmQpo2o8NMHZb9Jfgc05VvG0x%2Fzpk4NwifrNGLFWk%3D&reserved=0) and

[https://journals.plos.org/plosone/s/file?id=ba62/PLOSOne_formatting_sample_title_authors_affiliations.pdf](https://eur02.safelinks.protection.outlook.com/?url=https%3A%2F%2Ftrack.editorialmanager.com%2FCL0%2Fhttps%3A%252F%252Fjournals.plos.org%252Fplosone%252Fs%252Ffile%253Fid%3Dba62%252FPLOSOne_formatting_sample_title_authors_affiliations.pdf%2F1%2F010f019593e6ecbc-9921ebc3-6552-4ce5-bf9e-bb85feb80e26-000000%2FkQdryO-XKWyoSfEaC9vTJ_VqnpgtroiGlgiETcU-H_s%3D202&data=05%7C02%7Chtariq%40bournemouth.ac.uk%7C7bc3187e8f01447e8ddd08dd80e78192%7Cede29655d09742e4bbb5f38d427fbfb8%7C0%7C0%7C638808454268687066%7CUnknown%7CTWFpbGZsb3d8eyJFbXB0eU1hcGkiOnRydWUsIlYiOiIwLjAuMDAwMCIsIlAiOiJXaW4zMiIsIkFOIjoiTWFpbCIsIldUIjoyfQ%3D%3D%7C0%7C%7C%7C&sdata=iWh22E%2BMI1wljB%2B6mJoal5YrXQiJvBdU2KoClh98suU%3D&reserved=0)

We have carefully reviewed the manuscript and ensured that it meets PLOS ONE’s style requirements, including file naming. Please let us know if you find any other style violations.

2. Please note that PLOS ONE has specific guidelines on code sharing for submissions in which author-generated code underpins the findings in the manuscript. In these cases, we expect all author-generated code to be made available without restrictions upon publication of the work. Please review our guidelines at https://journals.plos.org/plosone/s/materials-and-software-sharing#loc-sharing-code and ensure that your code is shared in a way that follows best practice and facilitates reproducibility and reuse.

Thank you for the note. This is not applicable as the manuscript does not include any author-generated code.

3. We note that the grant information you provided in the ‘Funding Information’ and ‘Financial Disclosure’ sections do not match.

When you resubmit, please ensure that you provide the correct grant numbers for the awards you received for your study in the ‘Funding Information’ section.

Thank you for pointing this out. We have reviewed and corrected the grant numbers to ensure consistency between the ‘Funding Information’ and ‘Financial Disclosure’ sections.

4. Thank you for stating the following financial disclosure:

“This study was funded by Bournemouth

University and Dorset Healthcare University

Foundation Trust through a match-funded PhD studentship awarded to H. Tariq”

Please state what role the funders took in the study.  If the funders had no role, please state: "The funders had no role in study design, data collection and analysis, decision to publish, or preparation of the manuscript."

If this statement is not correct you must amend it as needed.

Please include this amended Role of Funder statement in your cover letter; we will change the online submission form on your behalf.

As requested, we have included the following statement in the cover letter:

*“The funders had no role in study design, data collection and analysis, decision to publish, or preparation of the manuscript.”*

5. We note that you have indicated that there are restrictions to data sharing for this study. PLOS only allows data to be available upon request if there are legal or ethical restrictions on sharing data publicly. For more information on unacceptable data access restrictions, please see [http://journals.plos.org/plosone/s/data-availability#loc-unacceptable-data-access-restrictions](https://eur02.safelinks.protection.outlook.com/?url=http%3A%2F%2Ftrack.editorialmanager.com%2FCL0%2Fhttp%3A%252F%252Fjournals.plos.org%252Fplosone%252Fs%252Fdata-availability%2523loc-unacceptable-data-access-restrictions%2F1%2F010f019593e6ecbc-9921ebc3-6552-4ce5-bf9e-bb85feb80e26-000000%2Fk1TwIFr6PI96NfTvOHPQCl7MY_Twk0x2gm4yfXXh2OY%3D202&data=05%7C02%7Chtariq%40bournemouth.ac.uk%7C7bc3187e8f01447e8ddd08dd80e78192%7Cede29655d09742e4bbb5f38d427fbfb8%7C0%7C0%7C638808454268695185%7CUnknown%7CTWFpbGZsb3d8eyJFbXB0eU1hcGkiOnRydWUsIlYiOiIwLjAuMDAwMCIsIlAiOiJXaW4zMiIsIkFOIjoiTWFpbCIsIldUIjoyfQ%3D%3D%7C0%7C%7C%7C&sdata=mCSBl1vdTydS8fuLONZ%2BGvfj3Ldxm41JHQGwQ8pIz70%3D&reserved=0).

Before we proceed with your manuscript, please address the following prompts:

a) If there are ethical or legal restrictions on sharing a de-identified data set, please explain them in detail (e.g., data contain potentially identifying or sensitive patient information, data are owned by a third-party organization, etc.) and who has imposed them (e.g., a Research Ethics Committee or Institutional Review Board, etc.). Please also provide contact information for a data access committee, ethics committee, or other institutional body to which data requests may be sent.

b) If there are no restrictions, please upload the minimal anonymized data set necessary to replicate your study findings to a stable, public repository and provide us with the relevant URLs, DOIs, or accession numbers. For a list of recommended repositories, please see

https://journals.plos.org/plosone/s/recommended-repositories. You also have the option of uploading the data as Supporting Information files, but we would recommend depositing data directly to a data repository if possible.

We will update your Data Availability statement on your behalf to reflect the information you provide.

Thank you for your guidance. We have now included an anonymised data set as Supporting Information to enable replication of our study findings. Full transcripts are not publicly available due to the small sample size and the inclusion of details about specific care homes, which could risk identifying the participants.

6. Please include captions for your Supporting Information files at the end of your manuscript, and update any in-text citations to match accordingly. Please see our Supporting Information guidelines for more information: [http://journals.plos.org/plosone/s/supporting-information](https://eur02.safelinks.protection.outlook.com/?url=http%3A%2F%2Ftrack.editorialmanager.com%2FCL0%2Fhttp%3A%252F%252Fjournals.plos.org%252Fplosone%252Fs%252Fsupporting-information%2F1%2F010f019593e6ecbc-9921ebc3-6552-4ce5-bf9e-bb85feb80e26-000000%2F1R1efL5Tgpt52SVPpIEukfnGVq76Fdy2oomaQrPJ-4k%3D202&data=05%7C02%7Chtariq%40bournemouth.ac.uk%7C7bc3187e8f01447e8ddd08dd80e78192%7Cede29655d09742e4bbb5f38d427fbfb8%7C0%7C0%7C638808454268926485%7CUnknown%7CTWFpbGZsb3d8eyJFbXB0eU1hcGkiOnRydWUsIlYiOiIwLjAuMDAwMCIsIlAiOiJXaW4zMiIsIkFOIjoiTWFpbCIsIldUIjoyfQ%3D%3D%7C0%7C%7C%7C&sdata=MMT83i6%2BN6wDqPtUZoUF4As4z3ho0NvYaZh9QR4G%2Fks%3D&reserved=0).

We have added captions for all Supporting Information files at the end of the manuscript and updated the in-text citations accordingly.

**Response to the Reviewers:**

|  | **Reviewer #1 comments** | **Author response** |
| --- | --- | --- |
| 1. | Sampling Justification: While the authors mention purposive sampling and the selection of 10 participants, a more detailed discussion on theoretical saturation and how it was determined would be beneficial | In the revised manuscript, we have elaborated on how saturation was determined during data collection and analysis (lines 136-140). |
| 2. | Interview Excerpts: Some interview excerpts are included, but the results section would benefit from a richer presentation of qualitative data, incorporating more quotes to illustrate the analyses. | Thank you for the valuable suggestion. We have included additional quotes to support our findings. Furthermore, an anonymised dataset has been added to the Supporting Information. |
| 3. | Discussion and Generalization: Some inferences regarding the applicability of ORACLE could be more cautious, avoiding broad generalizations based on a relatively small sample. | We have revised the discussion and conclusion to be more cautious in tone regarding the broader applicability of ORACLE. |
| 4. | Data Availability: Consider providing an anonymized dataset or additional transcripts as supplementary material. | Selected anonymised excerpts from participant interviews are included in the Supplementary Information. Full transcripts are not publicly available due to the small sample size and the inclusion of details about specific care homes, which could risk identifying participants. |
| 5. | Text Flow: Minor refinements in conciseness and organization, particularly in the introduction and discussion, would enhance clarity and impact. | We have reviewed the introduction and discussion sections and made revisions to improve conciseness and clarity. |
|  | **Reviewer #2 comments** | **Author response** |
| 1. | General: For the most part the paper is well written however in parts there is repetition of words and points that need to be removed in a revision. For example, page 3 lines 47-54 refers to immobility and a vicious cycle twice. | This has been reviewed and corrected. |
| 2. | Abstract: The methods could include that this was a partly deductive and pragmatic qualitative approach and also identify which country the work was undertaken. | We have updated the abstract (lines 20–21) as per your suggestion. |
| 3. | Introduction: The policy issues are highlighted later in the paper - this could be part of scene setting in the introduction that in England there is lack of attention (regulatory and clinically) compared to other aspects of care prior to the point that there is lack of a standard definition and measure of contractures. It would be useful to know if this is a national or international issue and if any previous attempts to address this gap as not clear currently. | We have revised the introduction (lines 95–101) to include the policy context. |
| 4. | Could add more about impact on quality of life for residents in the introduction if any evidence, literature in relation to this as the emphasis tends to be more on the physical impact of contractures. | We have revised the introduction (lines 69–74) to include evidence on the impact of contractures on residents’ psychological well-being and quality of life, to provide a more holistic context. |
| 5. | There is an assumption of clinical knowledge that not all readers may have so be beneficial to provide further explanation e.g. extremities - does this mean arms, legs, hands, feet? | Thank you for highlighting this. We have revised the text (lines 82–85) to replace “extremities” with the clearer term “limbs” (arms, hands, legs, and feet) to enhance understanding for all readers. |
| 6. | Useful to have a box with more description of ORACLE either in intro when mentioned or in methodology section. | We have added a descriptive box (Table 1) for ORACLE in the methodology section. |
| 7. | Methodology: Add where this was - England. also info on the CQC ratings of homes - all good?  Page 6 line 102 - data saturation not theoretical saturation  Page 6 line 107-108 - state not named homes but have in the acknowledgements unless pseudo-anonymised so suggest that provide more generalised thanks especially if do not have consent from homes to be named | We have specified the study was conducted in England (line 114) and added that all participating care homes had good CQC ratings (line 126).  This has been corrected.  We have revised the acknowledgement section to provide a generalised thanks to the care homes (lines 680-681). |
| 8. | Semi-structured interviews section: reference needs to be made to taking field notes as referred to in the analysis section but no detail given about this in the data collection. | We have added a brief description of taking field notes during interviews in Semi-Structured interviews section to align with the analysis details (line 163-165). |
| 9. | Data analysis: Braun & Clarke advise that themes are generated/identified - they do not 'emerge' see their recent papers on this. There is some conflation between codes and themes that needs to be addressed in this section. Were there any disagreements between the team that needed to be resolved? | We have made changes throughout the paper to align with Braun & Clarke’s guidance on theme generation/identification and to clarify the distinction between codes and themes. Additionally, we have added how team disagreements were resolved (lines 190-191). |
| 10. | A short explanation of researcher reflexivity would be beneficial. | We have added a short explanation of researcher reflexivity to the methodology section (lines 192-200). |
| 11. | Was there any PPIE (patient and public involvement and engagement)? | Thank you for highlighting this. We have added a brief section on Patient and Public Involvement and Engagement (PPIE) to the methodology section (lines 201-207). |
| 12. | Results: The three themes generally make sense. It could be made clearer which are more deductive/descriptive and those which are more inductive and conceptual. | We have clarified within the Results section which themes are more deductive and which are inductive (lines 223-228). |
| 13. | The use of quotes to demonstrate the themes is generally good. Some sub-themes lack quotes which could be added. | We have added the supporting quotes to the sub-themes that lacked them. |
| 14. | The section on acceptability and impact on residents is very brief and either needs quotes or suggest that this point is made in the description of the overall theme or in the discussion. | Thank you for your comment. Participants were asked to report any negative impact on residents during their ORACLE observations, which was a yes/no question; therefore no proper quotations are available for this sub-theme. |
| 15. | The paper lacks a discussion section and this could be created from some of references to literature in the results section and the points made in the implications so the conclusion should be a more succinct section. | Thank you for the suggestion. We have drafted a separate Discussion section by drawing on the literature cited in the results and your recommendations. We have also revised the conclusion to be more succinct. |
| 16. | Limitations: were all the staff nursing staff? | Thank you for your question. No nursing staff were available for interview, which we have acknowledged as a limitation. The participants were either senior staff members (either care managers or care coordinators) who organised the ORACLE assessments or Healthcare assistants (HCAs) who carried out the assessments. |
| 17. | References: Ensure all references are correct and complete as noted some are not. | We have reviewed and corrected all references to ensure they are complete and accurate. |
